# Greater physical fitness (Vo2Max) in healthy older adults associated with increased integrity of the Locus Coeruleus-Noradrenergic system

**DOI:** 10.21203/rs.3.rs-2556690/v1

**Published:** 2023-02-07

**Authors:** Emanuele RG Plini, Michael C. Melnychuk, Ralph Andrews, Rory T. Boyle, Robert Whelan, Jeffrey S. Spence, Sandra B. Chapman, Ian H. Robertson, Paul M. Dockree

**Affiliations:** Trinity College Dublin; Trinity College Dublin; Trinity College Dublin; Massachusetts General Hospital, Harvard Medical School; Trinity College Dublin; The University of Texas at Dallas; The University of Texas at Dallas; Trinity College Dublin; Trinity College Dublin

## Abstract

Physical activity (PA) is a key component for brain health and Reserve, and it is among the main dementia protective factors. Robertson proposed that the upregulation of Locus Coeruleus-noradrenergic (LC-NA) system might be a key aspects for building reserve and resilience to neurodegeneration. PA elicits an enhanced catecholamine response, in particular Noradrenaline (NA). By increasing physical commitment, greater amount NA is synthetised in response to higher oxygen demand. More trained individuals show greater capabilities to carry oxygen resulting in greater Vo2max (a measure of oxygen uptake and physical fitness indicator). In the current study, we hypothesised that greater Vo2 max could be related to greater LC-NA MRI integrity. As hypothesised, greater Vo2max related to greater LC integrity across 41 healthy adults (age range 60–72). As a control procedure, when the analyses were repeated for the other neuromodulators’ seeds (Serotonin, Dopamine and Acetylcholine) weaker associations emerged. The newly established link between Vo2max and LC-NA system offers further understanding of the neurobiology underpinning Reserve in relationship to PA. While this study supports Robertson’s theory proposing the upregulation of the noradrenergic system as a possible key factor building Reserve, it also provide ground for increasing LC-NA system resilience to neurodegeneration via Vo2max enhancement.

## Introduction

It is estimated that 40% of dementia causes are modifiable by simply addressing people’s lifestyle (Livingston et al. 2020). Of this 40%, lifetime physical activity (PA) has a crucial role in protecting against neurodegeneration and related cognitive decay (Livingston et al. 2020). It is documented that individuals with a greater physical fitness (PF) level can sustain more severe neuropathological burdens resulting in mitigated cognitive impairment (Livingston et al. 2020; Wallace et al. 2019; Erikson et al. 2018). For these reasons PA has been associated with the construct of reserve, which is defined as the individual’s ability to maintain better-than-expected cognitive and brain functions given brain insult or psychological and neurological illnesses (Stern 2000; Stern et al. 2020; Cabeza et al. 2018).It has become a generally accepted fact, both in the research community and the popular media, that PA and PF are both significant contributors to maintaining healthy cognition and reducing the risk of neurodegeneration (Song et al. 2022; Livingston et al. 2020; Wallace et al. 2019; Erikson et al 2018).

An obvious question to ask is what are the specific neurophysiological mechanisms underlying this relationship between PA and PF, and the preservation of brain health and cognitive function? In the current study we investigated whether the association between physical fitness and resilience to dementia may be partially explained by the noradrenergic theory of cognitive reserve (Robertson 2013&^2014^), proposing that continuous activation (and the related integrity) of the Locus Coeruleus (LC) – noradrenergic system could be a key candidate affecting cognitive reserve and resilience capabilities. Specifically, it is hypothesized that the continuous upregulation of the LC-NA system over the lifespan might be one of the key neurobiological components for building cognitive reserve, given the marked noradrenergic decay observed in neurodegenerative diseases (Braak et al. 2011; Mather et al. 2016). In fact, it is documented that the LC-NA system is the first site in the brain showing biomarkers of neurodegeneration, and its loss of functionality and integrity has been associated with cognitive decline (Braak et al. 2011; Ehrenberg et al. 2017; Betts et al. 2017; Satoh et al. 2019; Dahl et al. 2020). In Robertson’s Reserve model, the noradrenergic up-regulation should be protective against the neurodegenerative dynamics due to the neuroprotective role of NA in brain (Heneka et al. 2002; Heneka et al. 2015). Supporting evidence has shown anti-oxidative (Troadec et al. 2005) and anti-inflammatory (Feinstain et al. 2002; Giorgi et al. 2020) properties of NA along with promotion of neurogenesis and synaptogenesis by increasing brain derived neurotrophic factor (BDNF) (Counts et al. 2010; Hassani et al. 2020; Traver et al. 2005; Mannari et al. 2008). Accordingly, in the last decades several studies have supported this model reporting that LC-NA system integrity was indeed related to greater brain and cognitive health (Wilson et al. 2013; Elman et al. 2021; Jacobs et al. 2021; Plini et al. 2021; Dahl et al. 2022), including better attentive and mnemonic functions both in healthy and clinical populations (Clewett et al. 2016; Dahl et al. 2019; Dutt et al. 2021; Plini et al. 2021; Prokopiou et al. 2022).

**Figure 1:**
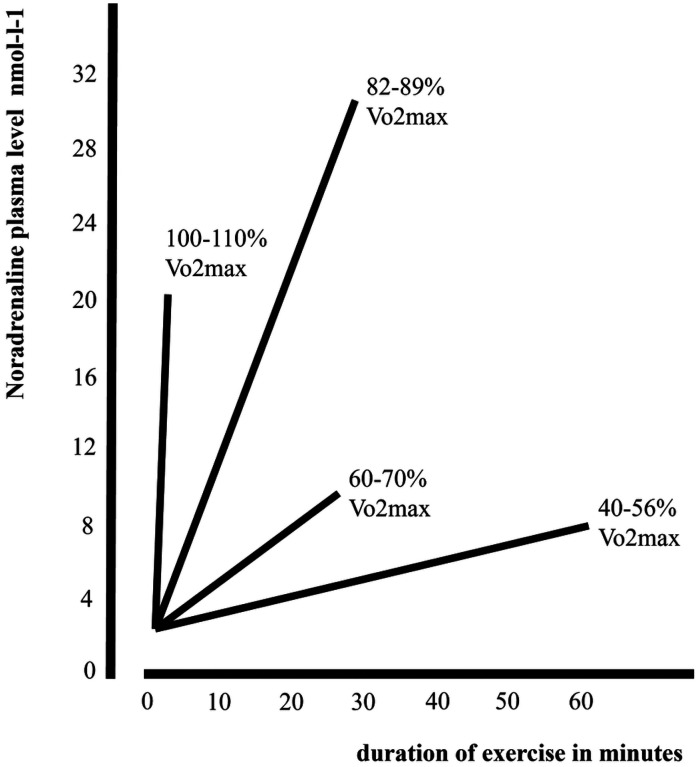
Plasma noradrenaline concentration in relation to intensity and duration of exercise. Figure adapted from Kjaer et al. 1987 with permission of Prof. Michael Kjaer

To date, no studies have directly investigated a possible relationship between LC-NA system integrity and PA. As shown in Fig. 1, by increasing physical exertion, a greater amount NA is synthesized and released in response to higher oxygen demand (Kjær et al. 1987; Bekar et al. 2012; Green et al 2007; Escourrou et al. 1984; Winter et al. 2007). Maximal training intensity (between 110% and 80% of maximal oxygen consumption – Vo2max) elicits the highest NA release in comparison with lesser demanding training intensities. Indeed, the LC-NA system is involved in breath cycles and LC neurons are highly sensitive to oxygen and Co2 variations (Melnychuk et al. 2021; Melnychuk et al. 2008; Biancardi et al. 2007). Further, trained individuals show greater oxygen carrying capacity while training (Crowley et al. 2022; Milanović et al. 2015). Vo2max is a measure of oxygen uptake commonly used as a main parameter of physical fitness and cardiovascular health (Strasser and Burtscher 2018; Hanscomb et al. 2021; Xu et al. 2022; Babu et al. 2022; https://health.ucdavis.edu/sports-medicine/resources/vo2description). A large meta-analyses carried out on 102.980 individuals found that greater Vo2 max was protective against all causes of mortality, while individuals with lower physical fitness show elevated risk (Kodama et al. 2009). More recently, greater levels of Vo2max have been associated with better brain and cognitive health (Wendell et al. 2013; Hwang et al. 2017; Pantzar et al. 2018; Wallace et al. 2019; Erikson et al. 2018), greater cortical thickness (Olivo et al. 2021) and greater white matter integrity (d’Arbeloff et al. 2020; Maleki et al. 2022). Furthermore, a study conducted on 2013 healthy adults (age range 21–84) found that greater cardio respiratory fitness (CRF) was associated with greater total brain volume, and greater grey matter volume in key brain regions such as the middle temporal gyrus, the hippocampal gyrus and the orbitofrontal cortex (Wittfled at el. 2020). In the same study greater Vo2 max was also related to greater volume in the bilateral anterior cingulate cortex, an area rich in noradrenergic receptors (Palomero-Gallager et al. 2015). Correspondingly, literature on dementia reported that higher Vo2 max and overall higher levels of physical fitness were associated with better brain and cognitive health (Manning et al. 2022; Nilsson et al. 2020; Bosch et al. 2020) and stronger resilience to neurodegeneration (namely more preserved brain integrity and functioning – Olivo et al. 2021; Petkus et al. 2021; Kurl et al. 2018; Ding et al. 2018; Livingston et al. 2020).

In light of the neuroprotective effects of greater Vo2 max together with the well documented LC-NA system role in dementia progression, we investigated whether variation in Vo2max was related to upregulation of the LC-NA system. We hypothesised that greater Vo2max would be associated with greater LC integrity (signal intensity) due to the neuroprotective effects of NA release. Specifically, greater Vo2max as an outcome variable of the frequent and intense LC-NA system up-regulation required by strenuous PA accumulated across the lifespan. (see Fig. 1 adapted from Kajer et al. 1987).

To test Robertson’s model, we performed voxel-based morphometry (VBM) analyses on 41 healthy older adults provided by the Centre for BrainHealth^®^, The University of Texas at Dallas -USA (Chapman et al. 2016). First, the relationship between LC signal intensity and Vo2 max was investigated. Second, the relationship between Vo2max and other key neuromodulator seeds were investigated as a control procedure. Therefore the analyses were repeated for the Dopaminergic system using the Ventral Tegmental Area (VTA), for the Serotoninergic system using on the Dorsal and Median Raphe (DR and MR) and on for the Cholinergic system using the Nucleus Basalis of Meynert (NBM). Third, we investigated the relationship between biological brain maintenance – BrainPAD (Boyle et al. 2021) and the neuromodulatory subcortical system to explore a possible relationship between Vo2max and BrainPAD. Our final aim was to investigate the relationship between LC and a measure of high order cognitive control assessed with the test of strategic learning (TOSL) (Chapman et al. 2016).

## Methods

### Participants, Neuropsychological tests and Vo2Max assessment

Data were provided by the Center of BrainHealth the University of Dallas Texas by Sandra Chapman and colleagues (Chapman et al. 2016). Participants were healthy older adults, 15 males and 26 females with age ranging from 60 to 72 years old. All participants underwent a preliminary screening, therefore none of them had no history of neurological or psychological illness and scored below 14 at the Beck Depression Inventory (BDI). In addition, all subjects were right-handed, not exceeded 40 of body mass index (BMI) and had as minimum education level an high school diploma. Lastly all participants’ IQ level was within the normative ranges and they scored above 26 at the Montreal Cognitive Assessment (MoCA). After the screening, participants underwent 3T high-res MRI and a neuropsychological battery which included the Test of Strategic Learning (TOSL) as primary measure for high order executive functioning. Indeed, TOSL is a tool sensitive to complex attention, memory and abstract reasoning. Subsequently, VO2max and maximum heart rate (MHR) were evaluated via electrocardiogram, a “*Lode—Excalibur Sport cycle ergometer, (Groningen, Netherlands) and a Jaeger Oxycon Pro, (Hoechberg, Germany)*” for Vo2max assessment. For detailed info please refer to (Chapman et al. 2016) and **see table 1**.

### Neuroimaging Processing And Analyses

41 3T T1-weighted high-res MRI images of healthy older adults (age range 60–72–15 males, 26 females) were processed using the standard CAT12 pipeline with a voxel size of 1mm3. The scans were modulated and oriented in MNI template and then whole brain images (MNI grey matter + white matter) were used for the formal analyses. In CAT12 a voxel-based morphometry (VBM) multiple regression model was built in order to assess the relationship between Vo2max and LC signal intensity. Age in years, gender, education, maxHR and total intracranial volume (TIV) were included as covariates and the positive relationship was tested in keeping with the main hypothesis, predicting that greater LC signal intensity would be related with higher scores Vo2 max. As a control procedure, the opposite, negative relationship was also tested. As additional control procedure, the same analyses were repeated for the neuromodulatory seed regions of the serotoninergic, dopaminergic and cholinergic systems, in order to contrast them with the noradrenergic hypothesis. The VBM analyses investigating the relationship between LC and BrainPAD and the TOSL test followed the same pipeline with the only exception being maxHR, which was not entered as a covariate. BrainPAD was calculated following the same procedure described in Boyle et al. 2021 (Boyle et al. 2021). BrainPAD is an objective index of brain maintenance based on the estimated degree of accelerated brain ageing. BrainPAD represents the difference between chronological age and biological age, which is predicted from grey matter density of the brain. It provides a candidate measure of brain maintenance in terms of years of accelerated or reduced brain degeneration. Therefore, higher discrepancies between the biological brain age and chronological age are indices of abnormal ageing and worse brain maintenance. For further information and greater details please refer to Boyle et al. 2021 and to Plini et al. 2021.

### Neuromodulatory Subcortical System

**Figure 2:**
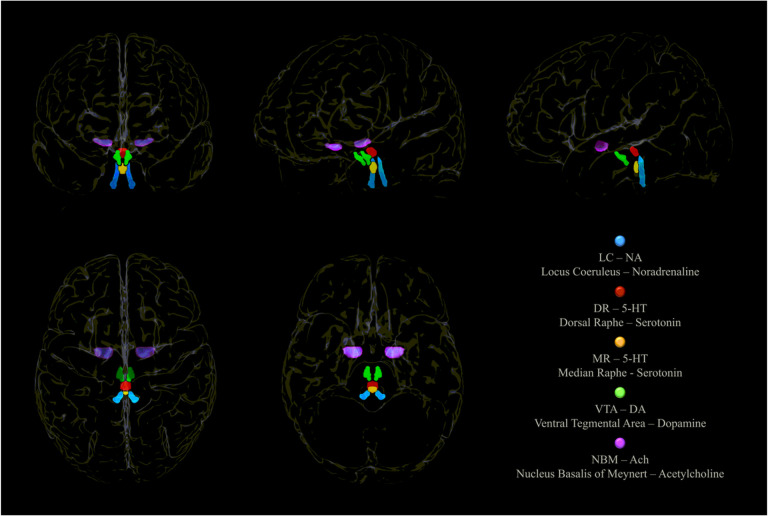
The neuromodulatory subcortical system: the main nuclei synthetizing and projecting to the cortex the main neuromodulators involved in cognition. The gure shows a 3D MRI reconstruction of the five neuromodulators’ seeds within the whole brain.

The isolation of the LC signal intensity was achieved using the “omni-comprehensive” LC mask (developed in our previous work – see Plini et al. 2021) which solves the inconsistent LC spatial localization reported by previous works (Keren et al. 2009 & 2015; Tona et al. 2017; Betts et al. 2017; Dahl et al. 2019; Liu et al. 2019; Rong Ye et al. 2020; Dahl et al. 2021; García-Gomar et al. 2022) without encroaching other pontine and cerebellar regions, and without crossing the walls of the 4th ventricles. The MRI LC omni-comprehensive mask was manually developed to carefully define a common space that included all the previous maps as to increase the likelihood of inclusion of the entirety of the LC. By doing so, structural analyses isolating the LC-NA system were made possible (further details can be found in supplementary materials of Plini et al. 2021 and at this link: https://www.youtube.com/watch?v=90bsA6Jqxs4). The other neuromodulatory seeds were based on previously published atlases: th MR and DR ROIs were provided by Beliveau et al. 2015, and the VTA mask was obtained by downloading the VTA MNI probabilistic map (Pauli et al. 2018) from the NeuroVault website (https://neurovault.org/ accessed on 15 December 2018). The NMB was developed on the basis of the probabilistic MNI maps of the acetylcholine cells of the Forebrain, which are provided by SPM Anatomy Toolbox 2.2c (https://www.fzjuelich.de/inm/inm1/EN/Forschung/_docs/SPMAnatomyToolbox/SPMAnatomyToolbox_node.html accessed on 15 December 2018) by Zaborszky et al. 2008 and George et al. 2011, Schulz et al. 2018, Liu et al. 2015, Kilimann et al. 2014, Koulousakis et al. 2019). These other neuromodulatory seeds were chosen on the base on their cortical and cerebellar projections. Consequently, the DR and the MR were chosen over the other Raphe nuclei because they vastly project to the cortex and the Cerebellum being the main serotoninergic nuclei of the CNS. Similarly, the VTA was chosen over the Substantia Nigra (SN) because the VTA is the main brain nucleus responsible of the cortical irroration of Dopamine (while the SN projects subcortically but not in the Cerebellum which does not shows relevant dopaminergic projections). Regarding the Cholinergic system, the NBM was chosen over the Tegmental Cholinergic Neurons, because it has the largest number of cholinergic neurons, and it projects diffusely to whole brain’s cortex (May and Paxinos 2012). Figure 2 shows the 3D MRI reconstruction of the five neuromodulators’ seeds.

### Bayesian Correlation Matrices And Mediation Analyses

Analyses were carried out in JASP (https://jasp-stats.org/). In a Bayesian regression model Vo2max was treated as dependent variable and the five neuromodulators entered as independent covariates, in order to compare their different effects against the “*null model*”. Age, gender, education, TIV and maxHR were treated as covariates and added to the “*null model*”. The analyses followed the default JASP setting with the exception being that bayesian information criteria (BIC) was selected in the advanced option of JASP interface.

Pearson’s and Bayesian correlation matrices were built to explore the different relationships between Vo2max, neuropsychological measures, BrainPAD with the five neuromodulators’ seeds. The average signal intensity of significant clusters of voxels produced by the VBM analyses was used in the analyses to better isolate the neuromodulators’ effect.

Mediation analyses with parallel multiple mediators was performed using Vo2max as a predictor (Y) and BrainPAD (X) as outcome. As multiple mediators, the five neuromodulators’ seeds were entered in parallel. The model was covaried for age, gender, education, TIV and maxHR. Standard model was set with 95% confidence intervals and 5000 bootstrap samples.

## Results

### Vo2max and Locus Coeruleus Integrity

**Figure 3:**
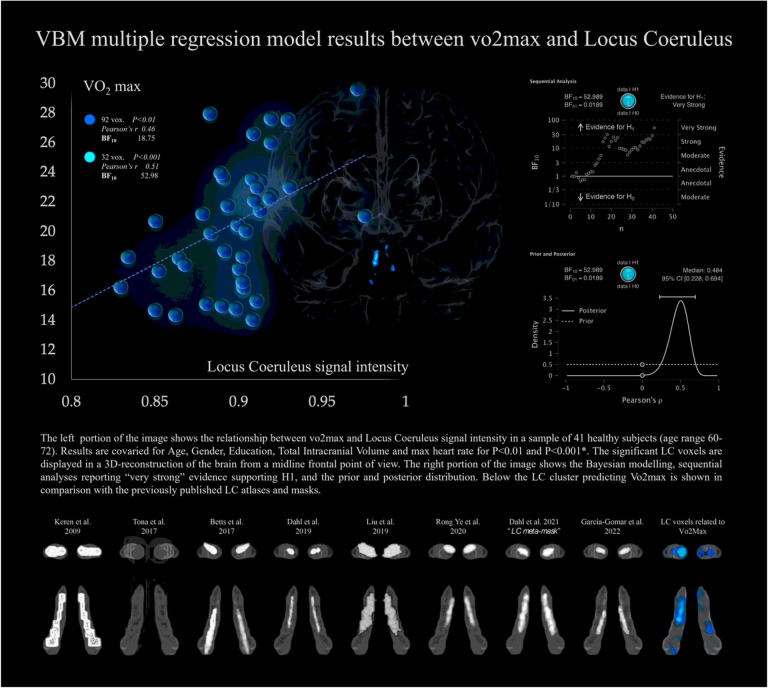
The left portion of the image shows the relationship between vo2max and Locus Coeruleus signal intensity in a sample of 41 healthy subjects (age range 60–72). Results are covaried for Age, Gender, Education, Total Intracranial Volume and max heart rate for P>0.01 and P>0.001*. The significant LC voxels are displayed in a 3D-reconstruction of the brain from a midline frontal point of view. The right portion of the image shows the Bayesian modelling, sequential analyses reporting “very strong” evidence supporting H1, and the prior and posterior distribution. Below the LC cluster predicting Vo2max is shown in comparison with the previously published LC atlases and masks.

As shown in **table 2**, the analyses confirmed a relationship between Vo2max and LC integrity after controlling for age, gender, TIV, education and maxHR. 92 voxels within the LC region significantly related with vo2max, i.e., greater LC signal intensity was associated with greater vo2max values. We note that a portion of the significant LC voxels in our findings overlaps the core of the previously published LC atlases and masks (Keren et al. 2009 & 2015; Tona et al. 2017; Betts et al. 2017; Dahl et al. 2019; Liu et al. 2019; Rong Ye et al. 2020; Dahl et al. 2021; García-Gomar et al. 2022), as shown in Fig. 3. The LC cluster survived when the statistical threshold was increased to p < 0.01 and p < 0.001 with a maximal Bayesian factor (BF_10_) of 55.98. Other weaker but relevant associations were observed between the DR and VTA signal intensity. A cluster of 32 voxel within the serotoninergic DR region and a cluster of 30 voxels within dopaminergic VTA region, which did not survive when the statistical threshold was raised to p < 0.001. Minor associations were found for the MR and NBM nuclei. When the inverse relationships were tested, negligible results were found and none survived at p < 0.01 threshold.

### Brain Maintenance (Brainpad) And Locus Coeruleus Integrity

The VBM analyses investigating the relationship between LC and biological brain maintenance and high order cognitive control revealed a similar pattern of findings. A significant relationship was found between LC signal intensity and BrainPAD; an LC cluster of 153 voxels was associated with greater biological brain maintenance. Weaker findings were observed for the NBM (52 voxels) and the DR (33 voxels). This pattern of findings replicate our previous findings relating biological brain maintenance primarily to the noradrenergic system in a larger sample of 686 subjects (Plini et al. 2021). The LC cluster survived the statistical threshold of p < 0.001. All the other ROIs showed a weaker pattern not surviving the p < 0.01 threshold (see table 4 in the supplementary materials).

### Neuropsychological Performance (Tosl) And Locus Coeruleus Integrity

In a similar vein, we observed a disproportionate link between LC and TOSL. Greater LC signal intensity related to higher scores on the TOSL, namely greater LC integrity was related with greater levels of high order cognitive control. A cluster of 101 voxels significantly predicted high order cognitive control performances, but did not survive multiple comparison corrections. Weaker and negligible results were observed for the other nuclei and none of them survived when the statistical threshold was increased to P < 0.01 (see table 5 in the supplementary materials). These findings are consistent with previous work relating LC signal intensity to better cognitive performances (Clewett et al. 2016; Dahl et al. 2019; Dutt et al. 2021; Plini et al. 2021; Prokopiou et al. 2022).

### Multiple Regressions, Mediation Analyses And Correlation Matrices

Correlation analysis revealed no relationship between the key variables investigated. As shown in **table 3**, the Bayesian multiple regression model including the group of five neuromodulators, showed that the combined effect of LC and VTA signal intensity is the best model relating to vo2max, followed by LC + VTA + DR. As stand-alone variable, LC is strongest predictor of vo2max followed by the VTA. These findings corroborate the VBM analyses and are consistent with our theoretical hypothesis linking the catecholaminergic involvement in physical activity to the subcortical neuromodulator’s MRI integrity.

Mediation analyses showed no mediation effect for the five neuromodulators and Vo2max / BrainPAD, however, a significant direct effect between vo2max and BrainPAD was observed, leading us to infer that physical fitness might affect biological brain maintenance (see table 6 in supplementary materials). No other significant effects were observed for TOSL.

## Discussion

In the current study we aimed to investigate a sample of 41 healthy adults to see whether vo2max was related to LC MRI signal intensity. As a secondary aim we investigated whether LC signal intensity could be related to a measure of biological brain maintenance (BrainPAD) and to higher order cognitive functions (TOSL) as already documented in literature (Plini et al. 2021). To this end, we tested the relationship between LC-NA system integrity and vo2max values and contrasted this hypothesis against the other main neuromodulatory systems. As we anticipated, greater vo2max values were related with greater LC signal intensity. We also observed weaker but significant relationships between Vo2max and VTA and DR signal intensity. Compared to the other neuromodulatory nuclei, greater LC integrity disproportionally related with higher physical fitness level, better biological brain maintenance and greater cognitive control abilities (TOSL). These findings are consistent with our proposal that vo2max might be used as a proxy index of the upregulation of the LC-NA system and its related integrity. The findings support the Noradrenergic theory of Reserve suggesting that greater physical activity, as indexed by higher oxygen uptake, may contribute reserve via the integrity of the LC/NA system. However, we also demonstrated a direct effect of greater vo2max upon biological brain maintenance independent of LC involvement.

Nevertheless, these preliminary findings linking Vo2max and the LC-NA system integrity are suggestive that greater fitness levels can affect LC integrity contributing to overall LC and brain health. Given the importance of LC integrity for predicting brain maintenance and cognition, vo2max should be further considered among the resilience factors to neurodegenerative diseases and possibly a key variable to target for curtailing or preventing LC-NA degeneration across the lifespan (Braak et al. 2011; Ehrenberg et al. 2017; Betts et al. 2017; Satoh et al. 2019; Dahl et al. 2020). Our interpretation of these findings is that greater noradrenergic tone elicited by greater physical fitness can contribute to the integrity of the LC-NA system via diverse mechanisms. One mechanism might involve the anti-inflammatory and anti-oxidative properties of NA, namely, increased NA release following physical exercise (Kjær et al. 1987; Winter et al. 2007) could reduce overall LC and brain inflammation in accordance with previous studies (Heneka et al. 2002; Heneka et al. 2012; Traver et al. 2005; Troadec et al. 2005; Feinstain et al. 2002). Similarly, increased NA might also increment the level of BDNF while reducing biomarkers of neurodegeneration within the LC and across the brain (Omulabi et al. 2021; Counts et al. 2010; Hassani et al. 2020; Traver et al. 2005; Mannari et al. 2008).

These neurobiological mechanisms might also explain the short- and long-term repercussion of physical activity on cognitive performances (Basso and Suzuki 2017; van Dogen et al. 2016; ; Blomstrand & Engvall 2020; Bosch et al. 2020; Nilsson et al. 2020; Winter et al. 2007), as several studies described enhanced cognitive functions immediately after physical exercise and better cognitive performances in well trained individuals (Wendell et al. 2013; Hwang et al. 2017; Pantzar et al. 2018; Wallace et al. 2019; Erikson et al. 2018). In a work from Nilsson and colleagues (Nilsson et al. 2020), the increased BDNF plasma levels following acute exercise were associated with greater cognitive training gains over a period of 12 weeks. Furthermore, another study by Engerof and colleagues (Engerof et al. 2018) found the regular physical activity led to greater bioavailability of BDNF resulting in greater brain volumes. These neurobiological processes are mediated by the LC-NA system which both underpin adaptation to physical stress and cognitive functioning (Kjær et al. 1987; Winter et al. 2007; Bekar et al. 2012; Green et al 2007; Jimenez et al. 2007; Escourrou et al. 1984; Robertson 2013&^2014^, Mather 2016). This interconnection was well presented empirically in a multimodal MRI study by Mather and colleagues (Mather et al. 2020), where several sets of isometric handgrip contractions for 18 seconds before cognitive tests were related to greater LC phasic activity during an fMRI attentional task, resulting in faster reaction times compared to control condition. In addition, Mather and colleagues, also reported that greater structural LC MRI contrast was related to better attentional control. Moreover, a previous study by Segal and colleagues (Segal et al. 2012) showed that 6 minutes of physical activity at 70% of vo2max enhanced memory consolidation of stimuli presented earlier both in MCI and healthy individuals. In parallel with enhanced memory, the groups which trained after stimuli presentation showed also significantly higher NA levels relative to controls. This interdependence between physical activity, cognition, catecholamines and BDNF levels was earlier demonstrated by Winter and colleagues (Winter et al. 2007) on a study on 27 healthy young subjects. Winter and colleagues measured catecholamines and BDNF plasma levels at baseline, after physical exercise and after a subsequent learning task. They found that, compared to rest- and moderate physical activity-conditions, only intense physical activity (6 min. running) elicited greater catecholamine and BDNF release and that these levels predicted greater immediate learning, and greater retention both at intermediate (1week) and long term (8–10months) stages. These studies, corroborate preceding evidence revealing 5-fold NA increase for 2 hours following exercise (Chatterton et al. 1996; Brenner et al. 1997; Brenner et al. 1998; Urhausen et al. 1995; Kjær et al. 1987), and well support the concept that enhanced NA release due physical activity might subsequently influence cognitive performance via LC-NA activity.

Our findings combined with the extant literature may indicate that vo2max variations can affect LC-NA integrity in humans, possibly influencing the degree of LC-NA resilience to neurodegeneration via several pathways including NA and BDNF release. Our preliminary findings show that vo2max varies with signal intensity, disproportionately within the structure of the LC, and highlights a potential mechanism in which physical fitness builds Reserve through the neuroprotective role of NA metabolism across the central nervous system (Robertson, 2013). However, it is worth mentioning that, as emerged both from VBM analyses and Bayesian modelling, our findings outline how other main neuromodulators can have a relevant role in relationship to vo2max and PA. Indeed, the observed associations between the dopaminergic VTA and serotoninergic DR and MR with vo2max are suggestive that greater physical fitness might be related with greater structural integrity of such nuclei. Indeed, similarly to NA but in minor extent, evidence shown increased 5-HT and DA following physical training (Heijnen et al. 2016; Cordeiro et al. 2017; Kjær et al. 1987; Bekar et al. 2012; Green et al 2007; Escourrou et al. 1984; Winter et al. 2007). Consistently, animal studies also shown greater concentrations both of 5-HT and DA within the cortex and the brainstem immediately after exercise (Heijnen et al. 2016; Dey et al. 1992; Meeusen and De Meirleir 1995; Foley and Fleshner 2008) implying greater integrity and functionality of such neuromodulatory systems in chronic (Foley and Fleshner 2008; Heijnen et al. 2016; Meeusen 2005; Dey et al. 1992). These findings together with our results are indicative that vo2max levels might affect 5-HT and DA nuclei integrity in humans for the same mechanisms discussed above. Nevertheless, the overall monoaminergic involvement in response to PA might be considered as factor capable to shape brain and cognitive health, and that greater Vo2max level can simultaneously stimulate Brainstem nuclei, possibly resulting in greater integrity.

## Limitations

The main limitation of the current preliminary study is the small sample size; a larger sample and a longitudinal design would provide more accurate casual relationships among these variables and would enable an understanding of possible trajectories across time. The number of participants may also explain why our significant associations did not survive multiple comparison corrections and the lack of mediation effects. However, our sample size is similar to other studies investigating vo2max in relationship to brain and cognitive variables (Manning et al. 2022; Nilsson et al. 2020; Bosch et al. 2020), and also matched the size of other relevant studies whose used the same methodology than ours (Olivo et al. 2021; Petkus et al. 2021). Furthermore, we have also enhanced the validity of our design by performing thorough control analyses, contrasting 9 different hypotheses to the main hypothesis, and systematically covarying for relevant confounding factors such as age, gender, education, total intracranial volume, and max heart rate. These control analyses and the employment of both null hypothesis statistical testing and Bayesian modelling, strengthens the reliability of our observations.

There are also methodological limitations of VBM analyses in retrospective studies which should be taken into account while considering these findings. Post-mortem histological investigations would offer more precise quantifications about the relationship between vo2max and the integrity of the neuromodulatory subcortical system. However, within these limitations, our findings are consistent with previous *in-vivo* evidence using similar methodology (Plini et al. 2021; Dutt et al. 2021; Olivo 2021; Petkus et al. 2021).

All in all, this is a preliminary investigation based on a relatively small sample focused on VBM analysis of circumscribed areas of the brainstem. Although, many of these findings are consistent with, and expand upon, the literature, further investigation in larger samples is required. Nevertheless, the current findings show consistency across multiple regression and Bayesian approaches.

## Conclusions And Clinical Implications

This is the first preliminary evidence linking *in vivo* LC-NA system integrity to Vo2max in healthy older subjects. These findings provide one possible neurobiological mechanisms connecting physical fitness to improved cognitive functions and resilience to neurodegenerative diseases. These novel and unique results shed light on possible neurobiological dynamics underpinning the relationship between physical fitness and brain health, whilst being consistent with Robertson’s theoretical framework positing the noradrenergic system as a key neuromodulatory basis of Reserve. Future studies, using physical exercise to specifically target the LC-NA system, should replicate the current findings focusing also on prospective trajectories including biomarkers, neuropsychological testing and other life-style factors. The current study provides additional evidence on the growing literature outlining the pivotal role of physical activity in matter of brain maintenance and cognitive health particularly in the face of neurodegenerative diseases (Matta Mello Portugal et al. 2013; Tyndall et al. 2018; Barnes et al. 2018; Livingston et al. 2020; Basso et al. 2022). This study adds support for preventative strategies focused on physical training protocols as a practical tool for increasing brain and cognitive health and protecting against neurodegeneration (Livingston et al. 2020; Batouli & Saba 2017; Freberg & Taglialatela 2022). Indeed, several studies reported that vo2max is effectively trainable using various methodologies at different ages in healthy populations (Gormley et al. 2008; Ozaki et al. 2013; Murawska-Cialowicz et al. 2015; Scribbans et al. 2016; Menz et al. 2019; Wen et al. 2019). Physical activity interventions also can significantly improve cognitive and brain health (Chapman et al. 2016; Kovacevic et al. 2020; Nilsson et al. 2020; Blomstrand & Engvall 2020; Zhu et al. 2021; Upadhyay et al. 2022; Basso et al. 2022), while evidence showed that in even MCI and early stages of dementia physical exercise can alleviate cognitive impairment, possibly slowing down disease progression (Anderson et al. 2011; Segal et al. 2012; Arcoverde et al. 2014; Reiter et al. 2015; Varma et al. 2017; Barnes & Corkery 2018; Yu et al. 2020).
